# A large-scale retrospective analysis reveals the fungal pathogen spectrum across diverse clinical specimens using metagenomic next-generation sequencing

**DOI:** 10.3389/fcimb.2026.1779223

**Published:** 2026-06-01

**Authors:** Shuo Zhang, Liangyu Li, Zhili Niu, Mengling Liu, Jieyu Mao, Jinwen Min, Sheng Xu, Ruiyun Li, Haiyue Zhang, Jing Yin, Xiaojun Wu

**Affiliations:** 1 Department of Pulmonary and Critical Care Medicine, Renmin Hospital of Wuhan University, Wuhan, Hubei, China; 2 Department of Respiratory and Critical Care Medicine, Shanghai Pulmonary Hospital, Tongji University School of Medicine, Shanghai, China; 3 Department of Clinical Laboratory, Institute of Translational Medicine, Renmin Hospital of Wuhan University, Wuhan, Hubei, China; 4 Department of Pulmonary and Critical Care Medicine, Fifth Hospital in Wuhan, Wuhan, Hubei, China

**Keywords:** clinical specimen, co- detection, epidemiology, fungal pathogen spectrum, invasive fungal disease, metagenomic next-generation sequencing

## Abstract

**Introduction:**

Early diagnosis of invasive fungal diseases (IFD) remains a major clinical challenge due to pathogen diversity and nonspecific symptoms. This study used metagenomic next-generation sequencing (mNGS) technology to comprehensively characterize fungal profiles across various clinical specimens and the demographic characteristics (sex and age) of the patient population. The results provide laboratory evidence to support the diagnosis and treatment of fungal infections.

**Methods:**

A total of 11,161 mNGS reports from clinical specimens collected at the Renmin Hospital of Wuhan University between March 2022 to August 2024 were retrospectively analyzed. Fungal spectra and patient demographics were comprehensively profiled and compared across different specimen types.

**Results:**

The highest fungal detection rate was observed in bronchoalveolar lavage fluid (36.85%, 1,985/5,387), followed by urine (22.76%, 264/1,160), blood (13.38%, 380/2,840), pleural and peritoneal fluid (12.91%, 174/1,348), cerebrospinal fluid (CSF) (13.82%, 17/123), and wound exudates (12.87%, 39/303). *Candida* species were the most frequently detected fungi across all specimen types except CSF, wherein *Aspergillus* predominated. Overall fungal detection rates were significantly higher in male patients than in female patients (26.76% vs. 23.84%, *P* < 0.01) and in individuals aged > 60 years compared with those aged ≤ 60 years (33.04% vs. 20.02%, *P* < 0.001), although this trend varied by specimen type. Multivariate logistic regression analysis confirmed that male sex (adjusted odds ratio [aOR]=0.893,95% confidence interval: 0.824-0.967, P = 0.006) and advanced age (≥80 years: aOR=14.77,95% confidence interval: 12.08-18.06, compared with minors) were independent risk factors for fungal detection. Among fungal-positive specimens, 68.28% (1,952/2,859) were co-detected with bacteria, and 15.63% (447/2,859) showed polyfungal detection (≥ 2 fungal species).

**Conclusion:**

In conclusion, our findings highlight the predominance of *Candida* and *Aspergillus*, identify elderly male patients as a high-risk population, and underscore the high frequency of bacterial-fungal co-detection. Overall, Clinicians should combine mNGS results with imaging, conventional fungal tests (G/GM assays, culture), and clinical presentation for a more accurate diagnosis of IFD.

## 1 Introduction

Invasive fungal disease (IFD) is a category of infectious conditions caused by the invasion of fungi into human tissues or the bloodstream, which triggers inflammatory responses and leads to organ dysfunction. The clinical diagnosis and management of IFD remain challenging owing to the diversity of fungal pathogens, often nonspecific and insidious clinical manifestations, and the lack of distinctive diagnostic features. Studies on the global disease burden have indicated that IFD represents an increasingly serious public health threat. A study has estimated that life-threatening fungal diseases affect more than 6.5 million people worldwide annually, resulting in over 3.7 million deaths, of which approximately 2.5 million are directly attributable to these infections (Denning, 2024). Early and accurate diagnosis is essential for initiating appropriate treatment and improving patient outcomes (Sedik et al., 2024).

Traditionally, the diagnosis of IFD has relied on clinical manifestations and supplementary laboratory or imaging examinations. However, the nonspecific nature of the clinical presentation often leads to delayed diagnosis. Auxiliary diagnostic methods include imaging studies, fungal culture, and non-culture-based techniques such as serological and immunological assays, such as the 1,3-β-D-glucan test, galactomannan assay and antigen/antibody detection, but each of these approaches has its own limitations, such as low sensitivity, slow turnaround time, and the potential for false-negative results due to prior antifungal therapy (Formanek and Dilling, 2019; Fang et al., 2023; Zhu et al., 2023). Consequently, the development of rapid and accurate diagnostic technologies is crucial to enable earlier interventions and improve clinical outcomes in patients with IFD (Kozel and Wickes, 2014).

In recent years, advances in molecular biology have revolutionized the diagnosis of IFD. Metagenomic next-generation sequencing (mNGS), with its superior diagnostic sensitivity and specificity (Yang et al., 2022a), is capable of rapidly and objectively detecting all pathogens using known genetic sequences. This technology is well suited for cases that are difficult to diagnose using conventional methods such as culture or serological testing (Chiu and Miller, 2019). In addition, mNGS does not require targeted amplification and can sequence resistance genes, thereby offering critical insights to guide clinical adjustment of therapeutic regimens (Han et al., 2019). Overall, the mNGS technology significantly enhances the accuracy and timeliness of etiological diagnosis, providing clinicians with crucial evidence for clinical decision-making.

In our previous analysis of patients with fungal detection in BALF by mNGS, only 12.9% of fungi-positive cases improved with antifungal therapy (Liu et al., 2024), this observation highlighted the need to first characterize the broader fungal detection landscape across different specimen types and populations. However, a few studies have systematically characterized the complete landscape of fungal pathogens across diverse clinical specimens using mNGS.

Considering the limitations of previous studies, this study aimed to characterize fungal profiles detected by mNGS across different specimen types and patient populations, testing whether detection patterns vary by specimen type, whether elderly men are at higher risk, and whether bacterial-fungal co-detection is common in respiratory samples. The findings of this study may provide robust evidence to support physicians in clinical diagnosis.

## 2 Materials and methods

### 2.1 Data source

This single-center, retrospective descriptive study included clinical specimens subjected to mNGS at Renmin Hospital of Wuhan University from March 1, 2022, to August 31, 2024. All enrolled samples were obtained from patients with clinically suspected infection, negative results from conventional microbiological testing, or failure of empirical anti-infective therapy, and met the clinical indications for mNGS testing. Specimens with incomplete reports and/or clinical information were excluded. Ultimately, 11,161 samples were included, encompassing six distinct specimen types: bronchoalveolar lavage fluid (BALF, n = 5,387), urine (n = 1,160), blood (n = 2,840), pleural and peritoneal fluid (n = 1,348), wound exudate (n = 303), and cerebrospinal fluid (CSF, n = 123). Data on patient age, sex, report date, department, clinical diagnosis, specimen type, and mNGS report details were collected. The study protocol was approved by the Ethics Committee of Renmin Hospital of Wuhan University (WDRM2025-K198).

### 2.2 mNGS analysis

DNA was extracted using the DNA Extraction Kit (Sansure Biotech Inc., Hunan, China) according to the manufacturer’s instructions. A DNA library was constructed using end-repair methods and overnight adapter ligation, followed by polymerase chain reaction amplification. Quality control analyzes were performed via high-throughput sequencing using a Nanopore MinION sequencer (Oxford Nanopore Technologies, Oxford, UK). Low-quality and duplicate sequences were removed, and high-quality reads were aligned to the human reference genome GRCh38 using Bowtie2 v2.4.3. The remaining reads were aligned against the National Center for Biotechnology Information (NCBI) GenBank database to annotate the pathogen genome. The reference databases included the 16S and internal transcribed spacer gene databases (downloaded from NCBI), which included 19,088 bacterial, 8,082 fungal, and 231 non-bacterial pathogen reference genes. mNGS-positive is defined as the detection of ≥20 sequences.

### 2.3 Statistical analysis

Categorical variables were compared between groups using Pearson’s chi-square test or Fisher’s exact test. To assess the strength of association, Cramer’s V was calculated as the effect size (V<0.1 indicates weak association, 0.1-0.3 indicates moderate association,>0.3 indicates strong association), and the odds ratio (OR) with a 95% confidence interval was reported. To control for confounding factors, a binary logistic regression was performed for multivariate analysis, with variables such as age, sex, specimen type, and department of origin included. Results were expressed as adjusted odds ratios (aOR) and 95% confidence intervals. All statistical analyzes were performed using IBM SPSS Statistics (IBM SPSS Statistics for Windows, version 26.0; Armonk, NY, USA). Two-sided *P*-values < 0.05 were considered statistically significant. Figures and tables were constructed using GraphPad Prism (v10.5.0).

## 3 Results

### 3.1 Fungal detection rates in overall and different specimen types

**Figure 1:**
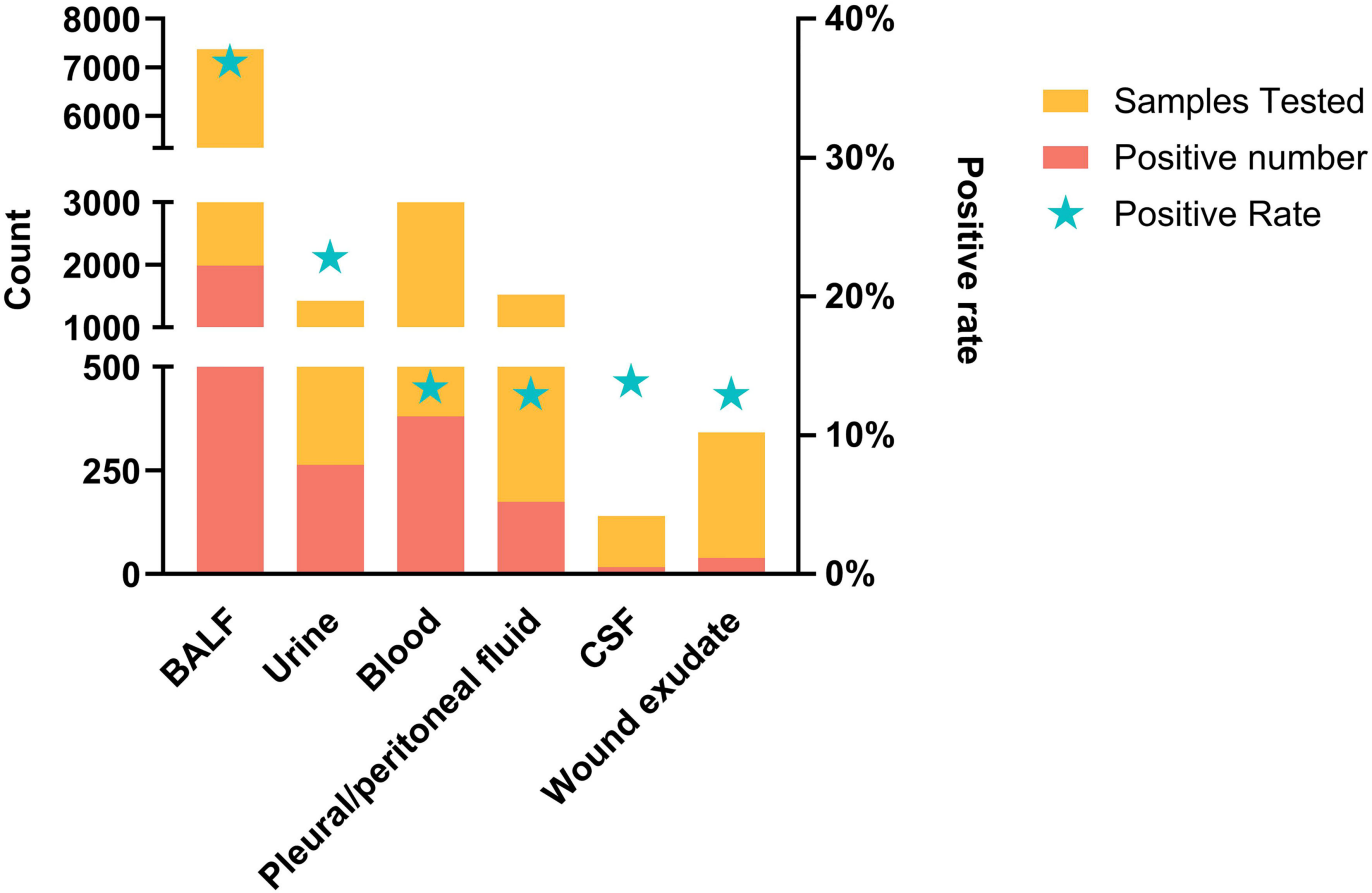
Number of positive specimens and fungal positivity rates across different specimen types. Bronchoalveolar lavage fluid (BALF): 36.85% (1,985/5,387); urine: 22.76% (264/1,160); blood: 13.38% (380/2,840); pleural/peritoneal fluids: 12.91% (174/1,348); cerebrospinal fluid (CSF): 13.82% (17/123); and wound exudate: 12.87% (39/303).

In total, 11,161 specimens were subjected to mNGS, of which 2,859 were positive for fungi, yielding an overall fungal detection rate of 25.62% (2,859/11,161). In terms of the number of positive specimens, BALF had the highest count (1,985 cases), followed by blood (380 cases), urine (264 cases), and CSF (17 cases). BALF exhibited the highest detection rate (36.85%), followed by urine specimens (22.76%). The detection rates among the remaining specimen types were relatively low and comparable at approximately 13% (**Figure 1**).

### 3.2 Differences in fungal species distribution across clinical specimens

A total of 103 distinct fungal species was identified among the 2,859 clinical specimens with positive mNGS results (fungal strains that could not be precisely classified at the species level were assigned to corresponding genera based on taxonomic principles). A total of 3,353 detections were recorded (with some specimens exhibiting co-occurrence of multiple fungi; see Section 3.6 for details). At the species level, the five most frequently detected fungi were *Candida albicans* (n = 929), *Candida tropicalis* (n = 257), *Aspergillus flavus* (n = 237), *Aspergillus fumigatus* (n = 191), and *Candida parapsilosis* (n = 181), which collectively accounted for 1,795 detections, representing 53.53% of all fungal detections. Among the 103 fungal species identified, 70.87% (n = 73) were detected less than 15 times (see **Supplementary Table 1** for detailed data).

**Figure 2:**
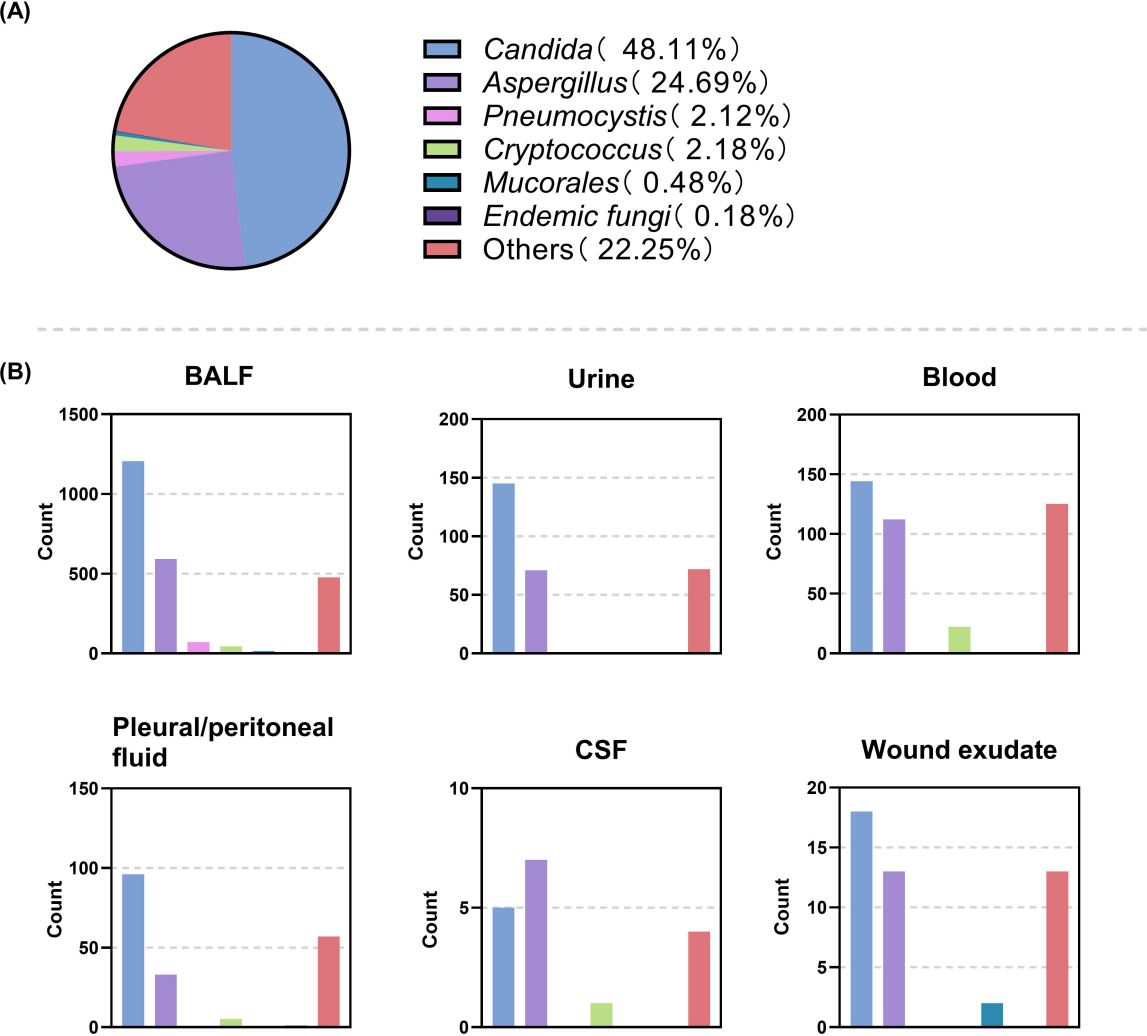
Distribution of fungal detections across genera and species. **(A)** Number of detected fungal species across different genera. **(B)** Distribution of fungal genera among fungal-positive specimens.

Based on the clinically relevant spectrum of invasive fungal pathogens, the fungi detected in this study were systematically categorized into seven major groups: *Candida* spp., *Aspergillus* spp., pneumocystis (specifically *Pneumocystis jirovecii*), *Cryptococcus* spp., Mucorales, endemic fungi, and other rare fungal genera (Sedik et al., 2024). At the genus level (**Figure 2A**), *Candida* (48.11%, n = 1,613) and *Aspergillus* (24.69%, n = 828) were the predominant fungal groups, both accounting for 72.80% of all identifications. Analysis of genus-level distribution across different specimen types (**Figure 2B**) revealed that *Candida* and *Aspergillus* exhibited broad specimen adaptability and were detected in all clinical specimen categories. In contrast, *Pneumocystis* was identified exclusively in BALF specimens. *Cryptococcus* (n = 73), Mucorales (n = 16), and endemic fungi (n = 6) were detected at lower frequencies and were primarily identified in BALF specimens.

### 3.3 Differences in fungal genera distribution across departments

The distribution patterns of different fungal genera across clinical departments were consistent. *Candida*, *Aspergillus*, *Cryptococcus*, and Mucorales were most frequently detected in the Department of Respiratory Medicine and Intensive Care Medicine (including the Department of Critical Care Medicine, Cardiac, Pediatric, and Acute Cardiovascular Care Intensive Care Units). Meanwhile, *Pneumocystis* was predominantly identified in the oncology and respiratory medicine departments. Endemic fungi were detected less frequently, with the highest occurrence observed in the Organ Transplantation Department. Detailed data are provided in **Supplementary Table 2**.

### 3.4 Sex-based characteristics of patients with fungal-positive specimens

**Figure 3:**
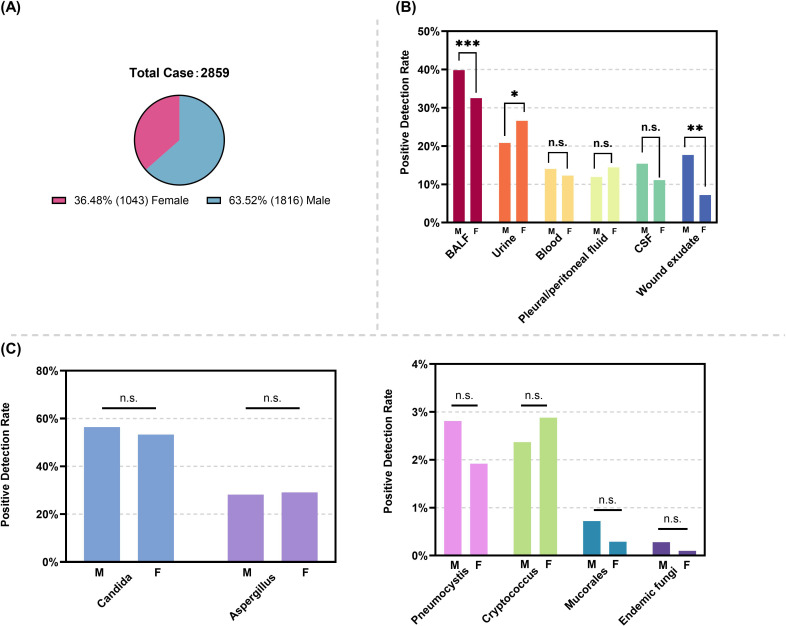
Sex-based differences in fungal positivity by specimen type and genus. **(A)** Number of males and females across all fungus-positive specimens. **(B)** Sex-based comparison of fungal positivity rates by specimen type. BALF: 39.84% vs. 32.53%; urine: 20.83% vs. 26.61%; blood: 14.04% vs. 12.29%; pleural/peritoneal fluids: 11.92% vs. 14.42%; CSF: 15.38 vs. 11.11%; and wound exudate: 17.68% vs. 7.19%. **(C)** Sex-based comparison of positivity rates by fungal genus. *Candida:* 56.44% vs. 53.31%; *Aspergillus*: 28.19% vs. 29.15%; *Pneumocystis*: 2.81% vs. 1.92%; *Cryptococcus:* 2.37% vs. 2.88%; *Mucorales:* 0.72% vs. 0.29%; and endemic fungi: 0.28% vs. 0.1%. ***P < 0.001; **P < 0.01; *P < 0.05; n.s., *P* > 0.05. M, male; F, female.

Among the 11,161 clinical specimens analyzed, the fungal detection rate was significantly higher in male patients than in female patients (26.76% [1,816/6,786] vs. 23.84% [1,043/4,375], respectively; χ²(1) = 11.91, *P* < 0.01, OR = 1.167 [95%CI: 1.069-1.275], Cramer’s V = 0.033) (**Figure 3A**).

Stratified analyzes based on specimen type (**Figure 3B**) showed that this sex-based difference in fungal detection rate remained statistically significant for BALF (χ² (1) = 29.86, *P* < 0.001, OR = 2.434 [95%CI: 2.160–2.742], Cramer’s V = 0.202), urine (χ² (1) = 4.91, *P* < 0.05, OR = 0.725 [95%CI: 0.546-0.964], Cramer’s V = 0.065), and wound exudate (χ² (1) = 7.38, *P* < 0.01, OR = 2.771 [95%CI: 1.298–5.915], Cramer’s V = 0.156) samples. In contrast, no statistically significant sex-based differences were observed for blood (χ² (1) = 1.75, *P* = 0.19), pleural and peritoneal fluid (χ² (1) = 1.80, *P* = 0.18), or CSF (χ² (1) = 0.44, *P* = 0.51).

Furthermore, to determine whether the detection rates of different fungal genera varied according to sex, comparisons were performed for *Candida*, *Aspergillus*, *Pneumocystis*, *Cryptococcus*, Mucorales, and endemic fungi. Chi-square tests (Fisher’s exact test for endemic fungi) revealed no statistically significant sex-based differences in detection rates for any of these fungal groups (*Candida*: χ² (1) = 2.63, *P* = 0.10; *Aspergillus*: χ² (1) = 0.29, *P* = 0.59; *Pneumocystis*: χ² (1) = 2.17, *P* = 0.14; *Cryptococcus*: χ² (1) = 0.69, *P* = 0.41; Mucorales: χ² (1) = 2.18, *P* = 0.14; and endemic fungi: Fisher’s exact test, *P* = 0.43), as shown in **Figure 3C**.

To further validate whether sex independently influenced fungal detection after adjusting for potential confounders (including age, specimen type, and department), a binary logistic regression analysis was performed. The results demonstrated that male sex remained a significant independent predictor of fungal detection (aOR = 0.893, 95% CI: 0.824-0.967, P = 0.006), indicating that the odds of fungal detection in females were approximately 10.7% lower than in males when other factors were held constant (**Supplementary Table 5**).

### 3.5 Age distribution of patients with fungal-positive specimens

**Figure 4:**
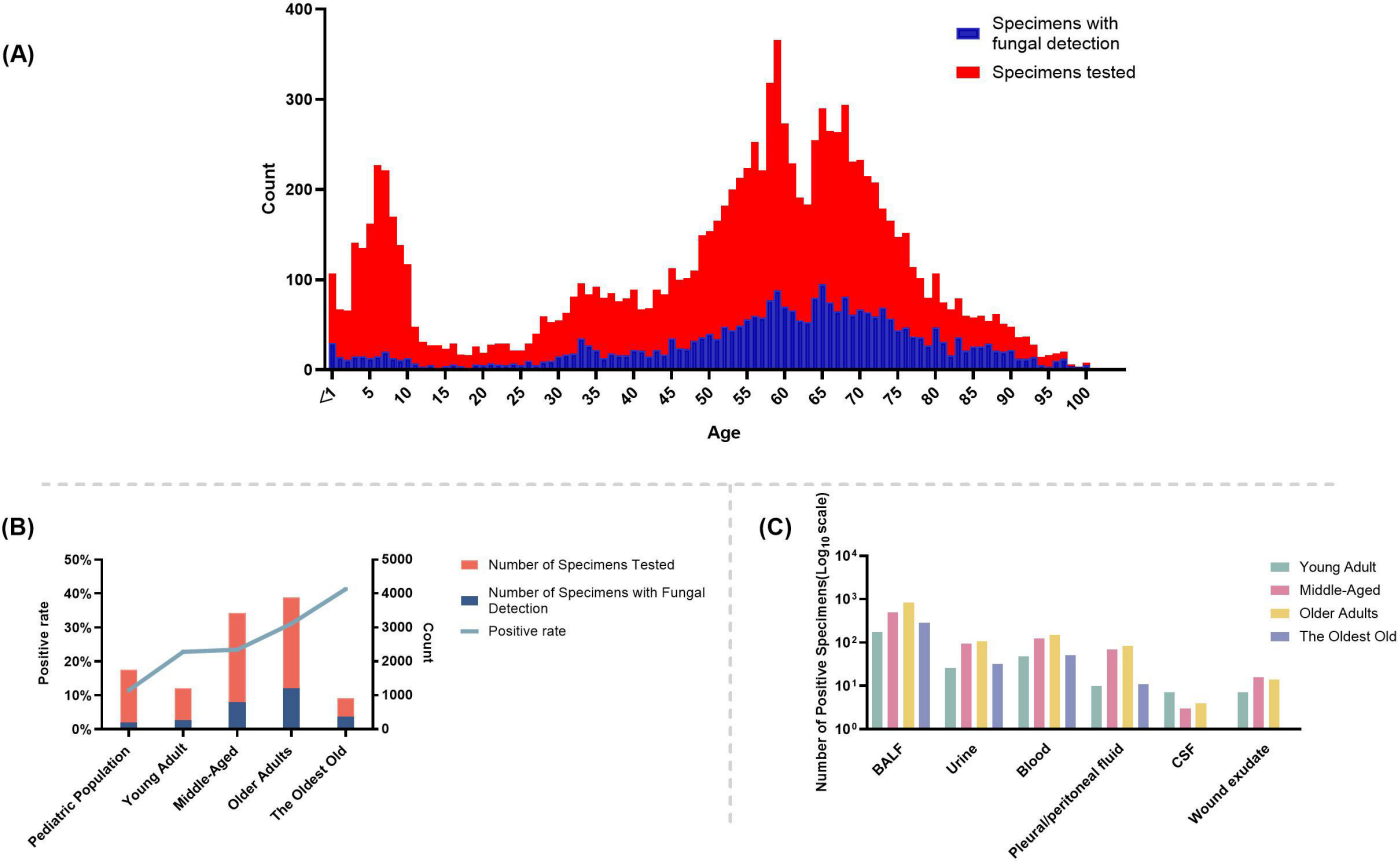
**(A)** Age Distribution Histogram. **(B)** Fungal positivity rates of specimens across different age groups. Pediatric population: 11.47% (201/1,753); young adults: 22.83% (274/1,200); middle-aged adults: 23.44% (801/3,417); older adults: 31.10% (1,207/3,881); and very old adults: 41.32% (376/910). **(C)** Age distribution of fungal detection across different specimen types. Y-axis shows log_10_-transformed specimen counts.

Patients were stratified into the following age groups: minors (<18 years), young adults (18–39 years), middle-aged adults (40–59 years), older adults (60–79 years), and very old adults (≥80 years). Fungal distribution across these age groups is shown in **Figures 4A, B**. Overall, the fungal detection rate was higher in individuals over 60 years of age than in those under 60 years of age (33.04% vs. 20.03%, χ² (1) = 242.89, *P* < 0.001,OR=1.970, [95%CI: 1.808–2.147], Cramer’s V = 0.148).

The age distribution patterns in BALF, urine, blood and pleural/peritoneal fluid specimens were consistent with those observed in the overall sample. In contrast, CSF specimens showed the highest proportion of positive cases among young adults, followed by older and middle-aged adults. For wound exudate specimens, middle-aged adults constituted the largest proportion of positive cases, followed by older and young adults.

Statistical analysis of these patterns revealed that the fungal detection rates in BALF and pleural/peritoneal fluid specimens were significantly higher in older adults (≥ 60 years) than in non-older adults (< 60 years) (*P* < 0.001). In contrast, no statistically significant differences between the two age groups were observed for urine, blood, CSF, or wound exudate specimens. The young adult group showed the highest number of fungal-positive CSF specimens and the middle-aged group had the highest number of positive wound exudate specimens, although these differences did not reach statistical significance (**Figure 4C**, **Supplementary Table 3**).

In terms of fungal genus distribution by age, the detection rate of *Candida* was higher in specimens from older adults (≥ 60 years) than in those from non-older adults (< 60 years) (57.57% vs. 53.63%, χ² (1) = 4.40, *P* = 0.04, OR = 1.174, [95%CI: 1.011–1.363], Cramer’s V = 0.039). In contrast, no statistically significant differences between the two age groups were observed for the detection rates of *Aspergillus*, *Pneumocystis*, *Cryptococcus*, Mucorales, or endemic fungi (**Supplementary Table 4**).

Multivariable logistic regression analysis, adjusting for sex, specimen type, and department, confirmed that age was an independent risk factor for fungal detection (**Supplementary Table 1**). Compared to minors (<18 years), the risk increased progressively with age: young adults (18–39 years: aOR = 7.64, 95% CI: 6.28-9.30), middle-aged adults (40–59 years: aOR = 8.39, 95% CI: 7.06-9.96), older adults (60–79 years: aOR = 10.25, 95% CI: 8.66-12.14), and very old adults (≥80 years: aOR = 14.77, 95% CI: 12.08-18.06), with all P-values <0.001. This graded increase in risk underscores age as a dominant factor in fungal positivity (**Supplementary Table 5**).

### 3.6 Pathogen co-occurrence in fungal-positive clinical specimens

**Figure 5:**
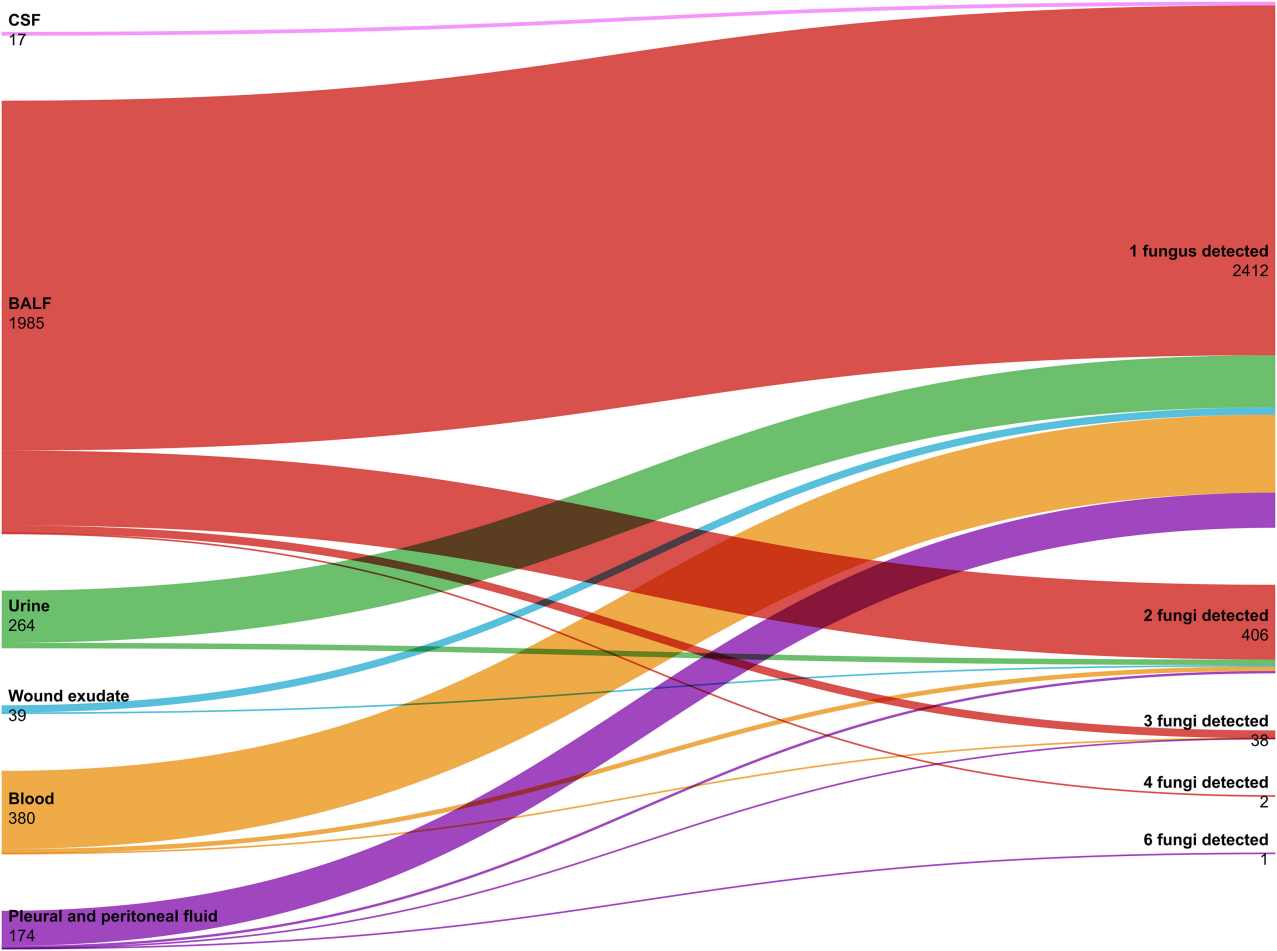
Number of fungi detected in fungal-positive specimens of different types.

Among the fungal-positive specimens, 83.37% (n = 2,412) were positive for a single fungal species, 14.20% (n = 406) for two fungal species, and 1.33% (n = 38) for three fungal species. Two BALF specimens were positive for four fungal species, and one peritoneal fluid specimen was positive for six fungal species. The distribution of polymicrobial fungi across different clinical specimen types is shown in **Figure 5**.

**Figure 6:**
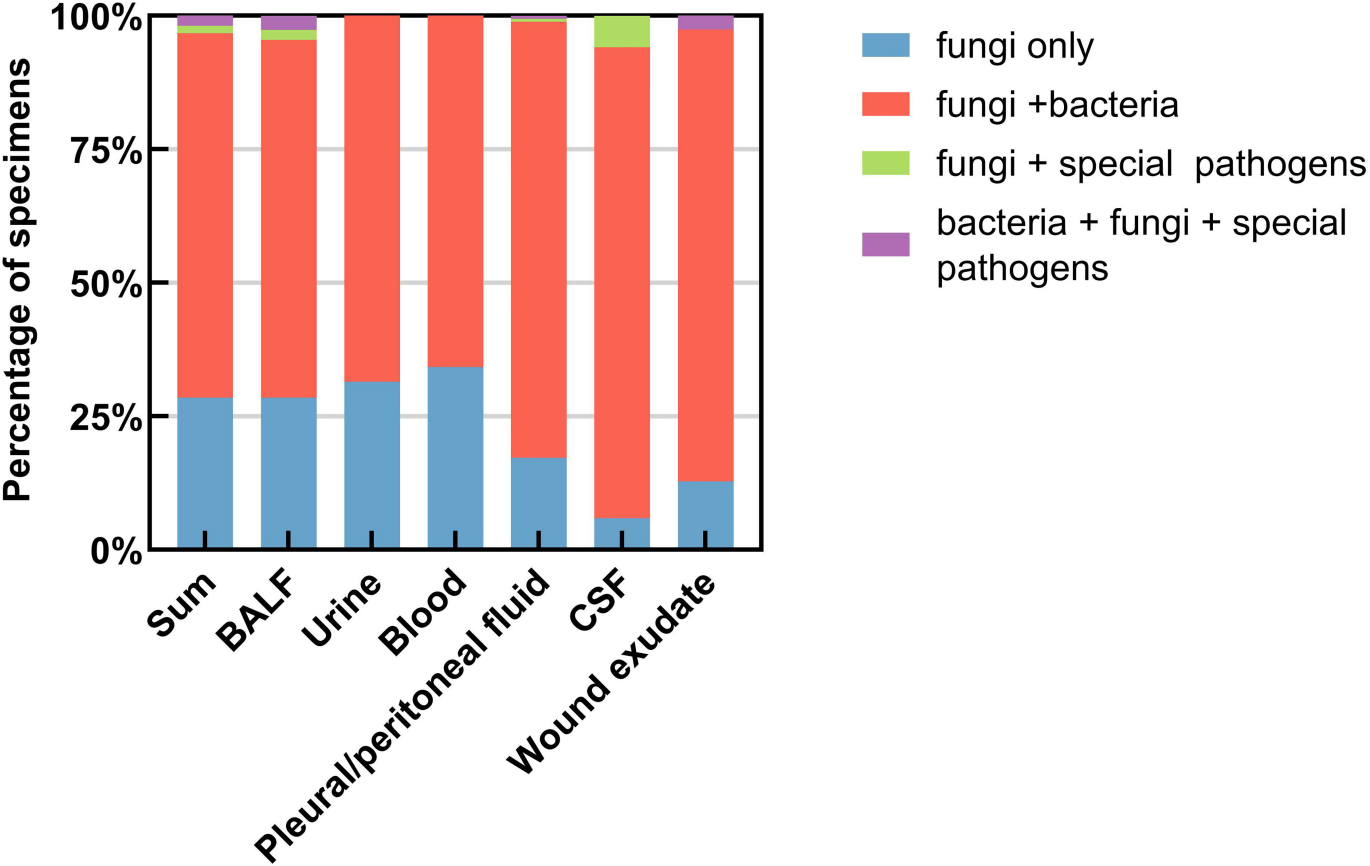
Percentage of specimens exhibiting different pathogen co-detection patterns, stratified by specimen type.

Among all submitted specimens, 68.28% (1,952/2,859) exhibited co-detection of fungi and bacteria, whereas fungi alone were detected in 814 specimens, accounting for 28.47% of all fungal-positive cases. Additionally, specimens with co-detection of fungi plus special pathogens and fungi plus bacteria plus special pathogens accounted for 1.33% (28/2,859) and 1.92% (55/2,859), respectively. The patterns of bacterial, fungal, and special pathogen co-detection across different clinical specimens are presented in **Figure 6**.

**Figure 7:**
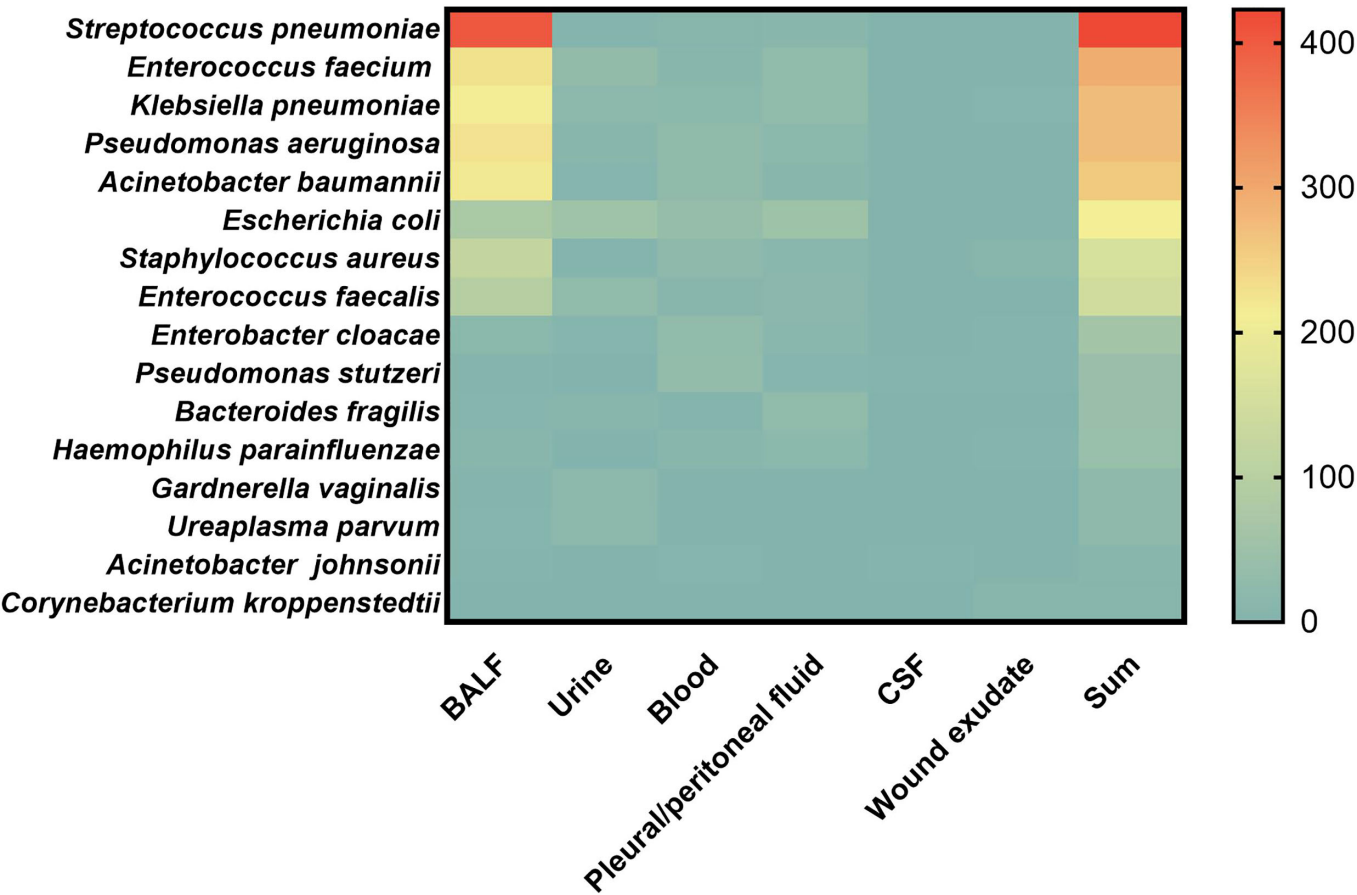
Summary of the top five bacterial taxa detected in different fungal-positive specimen types. Complete numerical data are provided in **Supplementary Table 6**.

A total of 209 distinct bacterial species were identified in the clinical specimens analyzed. Most bacterial species were detected at low frequencies, with 166 species (79.62% of all bacterial species detected) detected no more than 10 times. The five most frequently detected bacteria were *Streptococcus pneumoniae* (n = 423), *Enterococcus faecium* (n = 293), *Klebsiella pneumoniae* (n = 275), *Pseudomonas aeruginosa* (n = 275), and *Acinetobacter baumannii* (n = 258), which together accounted for 42.71% of all bacterial detections. The bacterial detection profiles of different clinical specimens are presented in **Figure 7**.

A total of 93 specimens (3.25% of all specimens) tested positive for special pathogens, including 79 cases of *Mycobacterium tuberculosis*, 11 of *Mycobacterium intracellulare*, 2 of *Mycobacterium xenopi*, and 1 of *Mycobacterium kansasii*. These special pathogens were predominantly detected in BALF specimens, accounting for 89 cases (95.70% of all special pathogen-positive specimens), comprising 75 cases of *M. tuberculosis*, 2 of *M. xenopi*, and 1 of *M. kansasii*. Additionally, *M. tuberculosis* was identified in two ascitic fluid specimens, one CSF specimen, and one wound exudate specimen.

## 4 Discussion

Through a large-scale retrospective analysis, this study is the first to systematically delineate the comprehensive landscape of fungal pathogens detected using mNGS across diverse clinical specimens. The key findings include the predominance of *Candida* and *Aspergillus*, highest detection rates in BALF and urine specimens, identification of male and older patients as high-risk populations for fungal positivity, and frequent occurrence of polymicrobial (bacterial + fungal) co-detection. These insights facilitate the early diagnosis and precise treatment of IFD, guide appropriate antifungal use, and optimize therapeutic strategies, as well as provide a solid scientific foundation for effectively controlling infections, improving patient prognosis, and reducing mortality. Therefore, this study has significant clinical value and translational relevance.

Our findings showed that *Candida* and *Aspergillus* were the most frequently detected fungi across all specimen types, collectively accounting for 72.80% of all identifications. Recent studies have demonstrated that in immunocompromised hosts, infections caused by these two genera constitute the main component of IFD and contribute substantially to incidence and mortality (Giannella et al., 2025; Chowdhary et al., 2023), which is consistent with our results. Although *Candida* species predominated across most specimen types, *Aspergillus* species were detected more frequently than *Candida* in CSF specimens. *Pneumocystis jirovecii* was identified exclusively in BALF specimens, consistent with its established role as a primary causative agent of *Pneumocystis* pneumonia in immunocompromised hosts and its low propensity for extrapulmonary dissemination (Lagrou et al., 2021). In contrast, although *Cryptococcus* most commonly affects the central nervous system and lungs (Chang et al., 2024), only one case was detected in the CSF specimens in this study, which may reflect the regional epidemiological characteristics of our sample sources. Regarding rare fungi, we detected 16 cases of Mucorales fungi in BALF, urine, and wound exudate specimens, as well as six cases of *Talaromyces marneffei* in BALF and ascitic fluid specimens. These fungi typically affect only specific immunocompromised individuals, such as those with AIDS (Steinbrink and Miceli, 2021; Wang et al., 2023). The detection of these rare fungi highlights the value of mNGS in enabling the diagnosis of rare fungal infections.

IFD is largely an “opportunistic infection,” and its onset is closely linked to the host’s immune status. Populations at risk of opportunistic fungal infections include organ transplant recipients, patients with hematologic diseases requiring stem cell transplantation (Elhaj Mahmoud et al., 2024), HIV/AIDS, or diabetes, and those on long-term immunosuppressive therapy (Fang et al., 2023). Our data indicate that although the distribution of fungal genera varied across departments, the overall detection was predominantly concentrated in units caring for immunocompromised populations, such as the intensive care, geriatrics, oncology, and organ transplantation departments. Owing to the high volume of specimens submitted and factors such as pulmonary fungal colonization, the Department of Respiratory Medicine remains the leading source of fungus-positive specimens.

Overall, the fungal detection rate in specimens obtained from male patients was higher than that in specimens from female patients. Egger et al. (2022) reported that with the exception of invasive candidiasis, nearly all fungal diseases exhibit a higher incidence in males, which is consistent with our findings. Multivariable logistic regression analysis confirmed that male sex was an independent risk factor for fungal detection, with females having an approximately 10.7% lower odds of fungal detection than males after adjusting for age, specimen type, and department (aOR = 0.893, 95% CI: 0.824-0.967, P = 0.006). Higher fungal detection rates were observed in BALF and wound exudate specimens from male patients, which is consistent with the overall trend. Interestingly, the detection rate in urine specimens was higher in female patients, which is strongly associated with anatomical and physiological factors such as the shorter, straighter urethra in females, its proximity to the reproductive tract, and hormonal levels (Timm et al., 2025; Yang et al., 2022b; Foxman, 2014; Neugent et al., 2022). Clinically, this finding suggests that for female patients with recurrent fungal detection in urine, attention should be paid not only to antifungal therapy but also to urogenital microbiota restoration and voiding habits. Furthermore, when interpreting mNGS reports of female urine specimens, clinicians should be aware of the higher likelihood of colonization to avoid overtreatment. No significant sex-based differences were observed in the detection rates of individual fungal genera. Notably, as the data collected in this study only indicated fungal detection rather than confirmed infection, whether sex-related differences exist in actual fungal infection rates remain unclear.

Invasive fungal infections have become an increasingly serious problem among older adults with an increasing incidence of opportunistic fungal infections (Kauffman, 2001). This is partly because older adult patients are more likely to undergo solid organ or bone marrow transplantation, receive aggressive treatments for malignancies, and take immunosuppressive medications for comorbidities (Baddley et al., 2011). Additionally, older patients are more vulnerable to invasive endemic or opportunistic infections and tend to experience worse outcomes (Kauffman, 2001). Overall, the fungal detection rate in specimens from the older adult population in our study was higher than that in specimens from non-older adults, consistent with the findings of previous studies. Notably, multivariable analysis revealed a striking age-dependent gradient in risk: compared to minors (<18 years), the adjusted odds of fungal detection increased to 7.6-fold in young adults, 8.4-fold in middle-aged adults, 10.3-fold in older adults, and nearly 14.8-fold in very old adults (≥80 years) (all P < 0.001). This dose-response relationship strongly supports age as a dominant driver of fungal positivity. However, further analysis revealed that not all specimen types exhibited age distribution patterns consistent with the overall trend; fungal detection rates in BALF and pleural/peritoneal fluid specimens were higher in the older adult group. Moreover, among the fungal genera detected, only *Candida* showed a higher detection rate in the older adult population, whereas other fungal genera did not exhibit age-related preferences.

The highest fungal detection rates were observed in BALF and urine specimens. Moreover, polymicrobial fungi were more frequently detected in BALF specimens. These discrepancies may be attributed to differences in the anatomical structures of the respiratory and urinary tracts, the characteristics of fungal colonization, and the clinical procedures used for specimen collection (Zinter et al., 2019; Gu et al., 2019; Langelier et al., 2018). This highlights a limitation of mNGS, namely its inability to distinguish between colonization and infection. Therefore, mNGS results should be interpreted in conjunction with clinical manifestations, other diagnostic findings, and epidemiological context and compared with no-template controls (Simner et al., 2018). In contrast, pathogen detection in normally sterile sites such as blood, CSF, and pleural/peritoneal fluid strongly suggests true infection. Although single-fungal detection was predominant across all specimen types and no cases of polymicrobial fungal detection were observed in CSF specimens, the co-detection of fungi remain a notable clinical consideration.

Wang et al. (2019) reported that mNGS exhibited significantly higher sensitivity than conventional methods for diagnosing mixed pulmonary infections, albeit at the expense of lower specificity. In the current study, nearly 70% of the fungal-positive clinical specimens exhibited pathogen co-detection. In BALF, urine, and blood specimens, which comprised the majority of the samples, the rate of bacterial-fungal co-detection was approximately 70%, whereas in the three specimen types with smaller sample sizes, co-detection rates increased to 80%. Although the interpretation of mNGS reports requires careful consideration of multiple factors and bacterial-fungal co-detection does not necessarily indicate true co-infection, several clinical insights warrant attention. Fungal infections often occur in immunocompromised individuals, and immunosuppression is a known risk factor for bacterial-fungal co-infection (Zhou et al., 2020). Moreover, antibiotic exposure has been shown to promote fungal overgrowth (Jain et al., 2021), and the clinical manifestations of bacterial-fungal co-infections tend to be more severe than those of the fungal infections alone. Therefore, in clinical practice, patients with suspected bacterial and fungal infections should be systematically evaluated for possible co-infection to facilitate the development of more precise antifungal therapeutic strategies.

### 4.1 Limitations and future perspectives

This study has certain limitations. First, due to the retrospective design, we lacked direct comparison with conventional microbiological methods (culture, serology) and clinical data (imaging, treatment response, mortality), precluding assessment of mNGS diagnostic performance and clinical utility. Therefore, our findings reflect a “fungal detection profile” rather than a “true infection profile,” and caution is warranted when extrapolating the findings to clinical diagnosis. Second, as a single-center retrospective study, the generalizability of our results may be inherently limited. Third, mNGS technology has inherent technical biases, including variations in DNA extraction efficiency, PCR amplification preferences, and database coverage, which may influence the relative detection frequencies of different fungal taxa.

Based on this study, future studies should: (1) prospectively integrate mNGS with full clinical data to distinguish infection from colonization; (2) expand to multicenter cohorts to validate generalizability and regional variations; (3) compare mNGS-guided versus empirical therapy for clinical utility and cost-effectiveness.

## 5 Conclusion

Through a retrospective analysis of mNGS results from 11,161 clinical specimens, this study systematically delineated the distribution characteristics of fungal pathogens. The findings revealed that: (1) *Candida* and *Aspergillus* species dominated across all specimen types; (2) BALF and urine specimens showed significantly higher fungal-positive detection rates compared with other normally sterile specimens; (3) higher fungal detection rates were observed in male patients and individuals aged over 60 years, although these trends were specimen type-dependent; multivariable analysis further confirmed that male sex (aOR = 0.893) and advanced age (≥80 years: aOR = 14.77) were independent risk factors for fungal detection; (4) approximately 70% of fungal-positive specimens exhibited co-detection of bacterial sequences while nearly 16% of specimens were positive for two or more fungal species. This study provides detailed data for understanding fungal detection profiles across diverse clinical specimens and offers epidemiological reference points for identifying high-risk populations and mixed-infection risks when interpreting mNGS reports in clinical practice. In general, clinicians should integrate mNGS results with specimen type (whether the specimen source belongs to a sterile site), host factors (including sex, age and immune state), imaging findings, conventional mycological tests (G/GM assay, culture), and the patient’s clinical presentation to accurately differentiate colonization from infection, thereby guiding precision antifungal therapy.

## Data Availability

The original contributions presented in the study are included in the article/**Supplementary Material**. Further inquiries can be directed to the corresponding author.
