# Supplementary material for: A large-scale retrospective analysis reveals the fungal pathogen spectrum across diverse clinical specimens using metagenomic next-generation sequencing
Source: Front Cell Infect Microbiol. 2026 Jun 1;16:1779223. doi: 10.3389/fcimb.2026.1779223 (PMC13265309; doi:10.3389/fcimb.2026.1779223)
Supplement: Supplementary file 1 [file DataSheet1.pdf]

Supplementary Material

1 Supplementary Tables

Table 1. Distribution of fungal detection events at the species level across different specimens.

Species \ Specimen	Bronchoalveolar lavage fluid	Urine	Blood	Pleural and peritoneal fluid	Cerebrospinal fluid	Wound exudate
Candida albicans	755	59	66	43	3	3
Candida tropicalis	183	26	29	14	0	5
Aspergillus flavus	214	3	14	5	0	1
Aspergillus fumigatus	179	2	5	2	1	2
Candida parapsilosis	120	14	22	17	1	7
Aspergillus sydowii	65	37	50	9	4	3
Alternaria alternata	96	12	38	6	0	3
Malassezia restricta	92	1	10	9	0	1
Aspergillus brocae	53	13	21	10	0	4
Candida glabrata	50	31	3	10	0	0
Yarrowia lipolytica	20	18	25	8	1	2
Pneumocystis jirovecii	71	0	0	0	0	0
Aspergillus spp.	41	0	14	0	0	0
Saccharomyces cerevisiae	43	3	2	4	0	0
Cladosporium cladosporioides	31	0	14	4	2	1
Aureobasidium pullulans	22	11	12	4	0	1
Clavispora lusitaniae	29	5	2	3	0	0
Cladosporium herbarum	26	2	3	1	1	0

Candida metapsilosis	19	4	5	2	0	1
Trichosporon asahii	17	4	5	4	0	0
Candida krusei	20	3	2	2	0	1
Cryptococcus neoformans	24	0	1	0	0	0
Cryptococcus curvatus	5	0	16	3	0	0
Penicillium citrinum	21	1	1	0	0	0
Aspergillus versicolor	14	2	5	1	1	0
Candida guilliermondii	2	1	10	2	1	1
Cryptococcus magnus	7	1	4	2	1	0
Rhodotorula mucilaginosa	8	3	0	4	0	0
Fusarium oxysporum	15	0	0	0	0	0
Candida orthopsilosis	12	0	2	1	0	0
Others	152	34	22	22	1	9

(1) This table above lists the top five fungi detected in each type of clinical specimen. (2) This table presents the number of events detected. When multiple fungal species were detected in a single specimen, each species was counted independently. Therefore, the sum of the numbers in each column may exceed the total number of positive specimens. The total number of detection events was 3,353.

Table 2. Distribution of fungal genera across different clinical departments.

Department \ Genera	***Candida*** **(sum = 1613)**	***Aspergillus*** **(sum = 828)**	***Pneumocystis*** **(sum = 71)**	***Cryptococcus*** **(sum = 73)**	***Mucorales*** **(sum = 16)**	***Endemic fungi*** **(sum = 6)**
**Respiratory Medicine**	403	284	17	22	3	1
**Critical Care Departments**	360	116	6	9	4	0
**Geriatric Medicine**	109	29	3	3	0	0
**Pediatrics (including Neonatology)**	53	71	6	6	1	0
**Oncology**	47	35	19	1	0	1
**Nephrology**	69	47	8	5	0	0
**Organ Transplantation**	16	17	3	7	0	2
**Thoracic Surgery**	12	11	2	3	0	1
**Gastrointestinal Surgery**	57	23	1	3	0	1

Note: Intensive Care Medicine includes Intensive Care Unit, Cardiac Intensive Care Unit, Pediatric Intensive Care Unit, and Acute Cardiovascular Care Center.

Table 3. Comparison of fungal detection rates in different clinical specimens between older and non-older adult groups.

Specimen type	Comparison groups	Statistical value	P value	Statistical conclusion
**BALF**	Older Adults vs. Non-Older Adults	χ^2 (1)= 219.23	< 0.001	Detection rate significantly higher in older adults
**Urine**	Older Adults vs. Non-Older Adults	χ^2 (1) = 0.00	0.95	No statistically significant difference
**Blood**	Older Adults vs. Non-Older Adults	χ^2 (1) = 0.35	0.55	No statistically significant difference
**Pleural and Peritoneal Fluids**	Older Adults vs. Non-Older Adults	χ^2 (1) = 13.77	< 0.001	Detection rate significantly higher in older adults
**CSF**	Older Adults vs. Non-Older Adults	Fisher's Exact Test	0.76	No statistically significant difference
**Wound Exudate**	Older Adults vs. Non-Older Adults	χ^2 (1) = 0.43	0.51	No statistically significant difference
**CSF**	Young Adults vs. All Other Age Groups	Fisher's Exact Test	0.12	No statistically significant difference
**Wound Exudate**	Middle-Aged Adults vs. All Other Age Groups	χ^2 (1) = 0.27	0.61	No statistically significant difference

Note: (1) Older adults were defined as individuals aged ⩾ 60 years. (2) Young adults were defined as individuals aged 18-39 years. (3) Middle-aged adults were defined as individuals aged 40-59 years. (4). Fisher's exact test was used when the expected cell count in the 2×2 contingency table was < 5.

Table 4. Comparison of detection rates of different fungal genera between older and non-older adult groups.

Genus	Comparison groups	Statistical value	P value	Statistical conclusion
Candida	Older Adults vs. Non-Older Adults	$\chi^2(1) = 4.3995$	0.04	Detection rate significantly higher in older adults
Aspergillus	Older Adults vs. Non-Older Adults	$\chi^2(1) = 0.2123$	0.65	No statistically significant difference
Pneumocystis	Older Adults vs. Non-Older Adults	$\chi^2(1) = 0.2429$	0.62	No statistically significant difference
Cryptococcus	Older Adults vs. Non-Older Adults	$\chi^2(1) = 0.1144$	0.74	No statistically significant difference
**Mucorales**	Older Adults vs. Non-Older Adults	$\chi^2(1) = 0.7934$	0.37	No statistically significant difference
**Endemic Fungi**	Older Adults vs. Non-Older Adults	Fisher's Exact Test	1.0	No statistically significant difference

Note: (1) The Older adults is defined as individuals aged ≥ 60 years. (2) Fisher's exact test was used when the expected cell count in the 2×2 contingency table was < 5.

Table 5. Multivariable logistic regression analysis of factors associated with fungal detection.

Characteristic	B	S.E.	Wald	P value	Adjusted OR	95% CI
**Sex**			7.697	0.006		
Male					1.000 (Reference)	
Female	-0.114	0.041	7.697	0.006	0.893	0.824 - 0.967
**Age Group (years)**			2828.623	<0.001		
<18					1.000 (Reference)	
18-39	2.033	0.100	411.114	<0.001	7.637	6.275 - 9.296
40-59	2.127	0.088	588.354	<0.001	8.388	7.064 - 9.961
60-79	2.327	0.086	729.719	<0.001	10.252	8.659 - 12.138
≥80	2.692	0.103	688.124	<0.001	14.767	12.076 - 18.057
**Specimen Type**			1482.098	<0.001		
BALF					1.000 (Reference)	
Urine	-2.187	0.066	1094.473	<0.001	1.466	0.099 - 0.128
Blood	-2.129	0.087	600.257	<0.001	0.112	0.100 - 0.141
Pleural/Peritoneal fluid	-2.228	0.162	189.124	<0.001	0.119	0.078 - 0.148
Wound exudate	-1.901	0.237	64.482	<0.001	0.108	0.094 - 0.238
CSF	0.383	0.137	7.860	0.005	0.149	1.122 - 1.916
**Department**			157.202	<0.001		
Respiratory Medicine						
ICU	0.709	0.072	97.936	<0.001	1.000 (Reference)	1.766 - 2.338
Urology	1.365	0.157	75.551	<0.001	2.032	2.878 - 5.326
Oncology	0.531	0.134	15.712	<0.001	3.915	1.308 - 2.211
Other Departments	0.661	0.066	98.839	<0.001	1.701	1.700 - 2.206
					1.936	
**Constant**	-2.588	0.095	747.578	<0.001		
					0.075	

Note: (1) Intensive Care Medicine includes Intensive Care Unit, Cardiac Intensive Care Unit, Pediatric Intensive Care Unit, and Acute Cardiovascular Care Center. (2) "Other Departments"

serves as the reference category, which includes departments not specified in the analysis (e.g., Organ Transplantation, Geriatrics, etc.).

Table 6. Ranking of bacterial species by frequency of co-detection with fungi.

Specimen type	Top 5 bacterial species (descending order)
**BALF**	*Streptococcus pneumoniae* (n = 402)
	Enterococcus faecium (n = 227)
	Klebsiella pneumoniae (n = 212)
	Pseudomonas aeruginosa (n = *225)*
	Acinetobacter baumannii (n = 218)
**Urine**	*Escherichia coli* (n = 53)
	Enterococcus faecium (n = 28)
	Enterococcus faecalis (n = 27)
	Gardnerella vaginalis (n = 17)
	Klebsiella pneumoniae (n = 16)
	Ureaplasma parvum (n = 16)
**Blood**	*Escherichia coli* (n = 32)
	Pseudomonas stutzeri (n = 30)
	Enterobacter cloacae (n = 27)
	Acinetobacter baumannii (n = 23)
	Pseudomonas aeruginosa (n = 23)
**Pleural and peritoneal fluid**	*Escherichia coli* (n = 51)
	Enterococcus faecium (n = 27)
	Bacteroides fragilis (n = 27)
	Klebsiella pneumoniae (n = 27)
	Pseudomonas aeruginosa (n = 15)
**CSF**	*Pseudomonas stutzeri* (n = 2)
	Acinetobacter johnsonii (n = 2)
**Wound exudate**	*Corynebacterium kroppenstedtii* (n = 6)
	Staphylococcus aureus (n = 6)
	Klebsiella pneumoniae (n = 4)
